# Understanding tobacco use disparities among Florida adolescents: The impact of sexual minority status and school-based violence victimization

**DOI:** 10.18332/tpc/195288

**Published:** 2024-11-20

**Authors:** Caleb M. Gumbs, Sandra Suther, Alana Steffen, Alicia K. Matthews

**Affiliations:** 1 Department of Medicine, University of Illinois, Chicago, United States; 2 College of Nursing, University of Illinois, Chicago, United States; 3 College of Pharmacy and Pharmaceutical Sciences, Institute of Public Health, Florida Agricultural and Mechanical University, Tallahassee, United States; 4 School of Nursing, Columbia University, New York, United States

**Keywords:** adolescents, tobacco use, school violence, sexual minority, substance use

## Abstract

**INTRODUCTION:**

Adolescent tobacco use remains a significant public health issue with long-term health consequences. This study investigates the relationship between sexual minority status, school-based violence victimization, and tobacco use among adolescents. The objective is to determine the prevalence of school-based violence victimization and tobacco use behaviors and identify key demographic and experiential risk factors.

**METHODS:**

Data were derived from the Florida Youth Risk Behavior Survey, collected biennially from 2013 to 2021. Participants included high school students who answered demographic questions on sexual orientation, race/ethnicity, sex, and grade. Bivariate analyses and binary logistic regression models examined associations between school-based violence and tobacco use, controlling for demographic factors.

**RESULTS:**

Of the 26510 participants, 15.8% identified as sexual minorities. Cigarette smoking was reported by 18.0% of the sample, with a higher prevalence among sexual minority students (27.3%) and students in 12th grade (22.5%). Sexual minority students reported higher rates of school-based violence, including being bullied at school (24.4%) and electronically bullied (22.6%). Overall, 29.3% of students experienced school-based victimization. Tobacco use was significantly associated with school-based violence (AOR=2.04; 95% CI: 1.91–2.19) with higher odds for sexual minority students (AOR=1.91; 95% CI: 1.75–2.09).

**CONCLUSIONS:**

The findings highlight a significant association between school-based violence and tobacco use among adolescents. Sexual minority students are at higher risk for both victimization and tobacco use. These results underscore the importance of violence prevention strategies and creating inclusive, supportive school environments that embrace sexual and gender diversity to mitigate these risks and promote overall student well-being.

## INTRODUCTION

Adolescent tobacco use remains a significant public health issue, affecting mental, physical, and educational outcomes in both the short- and long-term. It increases the likelihood of developing chronic health conditions later in life^1-5^. Despite efforts to reduce cigarette smoking, the rising popularity of electronic vapor products has created new challenges^6,7^. In 2019, a total of 50.1% of US high school students had ever used electronic vapor products, and 24.1% had ever tried cigarette smoking. Current electronic vapor product use was 32.7%, while current cigarette smoking was 6.0%^5^. Studies indicate that a substantial number of high school students continue to use various tobacco products, emphasizing the need for innovative and sustained efforts in tobacco prevention and control among youth^5-7^. Given the profound short- and long-term consequences, it is crucial to understand the factors that contribute to tobacco use among adolescents, to develop effective prevention and intervention strategies.

Research has identified a range of risk factors that influence tobacco use among adolescents, including demographics such as age and gender, family history of tobacco use, peer influences, and experiences of trauma^8-12^. Exposure to violence, particularly school-based violence, significantly increases the risk of tobacco use among adolescents^12,13^. For instance, studies have shown that adolescents who experience bullying are more likely to use tobacco compared to those who are not victimized^13,14^. The relationship between school-based violence and tobacco use is complex, with such experiences often serving as a trigger for starting or escalating tobacco use^8,12,15^. Additionally, the impact of school-based violence on tobacco use may vary depending on the frequency and severity of the violence encountered^12,16^.

The minority stress theory offers a valuable framework for understanding how stressors related to marginalized identities, such as race, ethnicity, sexual orientation, and gender, impact health outcomes, including tobacco use behaviors^17,18^. Adolescents from marginalized backgrounds often face unique stressors like discrimination, rejection, and internalized stigma, which can heighten their vulnerability to adverse health outcomes^17^. The interaction of these identities with other risk factors, such as school-based violence and trauma, may amplify these stressors, leading to increased tobacco use as a coping mechanism^17^. Research indicates that sexual minority adolescents are significantly more likely to experience school-based violence victimization than their heterosexual peers^13,17-19^. Additionally, marginalized adolescents often encounter barriers to accessing appropriate support and resources, further increasing their likelihood of using tobacco as a form of self-medication^17^.

Given these complexities, this study aimed to explore the relationship between sexual minority status, school-based violence victimization, and tobacco use. Specifically, the study sought to determine the prevalence and risk factors of tobacco use behaviors. By analyzing secondary data sources, this research aimed to provide a more detailed understanding of how these variables intersect and influence tobacco use patterns. The study hypothesized that adolescents who experience school-based violence victimization would have higher rates of tobacco use compared to their peers who do not use tobacco.

## METHODS

### Instrument

This study utilized data from the Florida Youth Risk Behavior Survey (YRBS), a cross-sectional secondary data source collected by the Florida Departments of Education and Health during the spring of 2013, 2015, 2017, 2019, and 2021. The CDC designed the Youth Risk Behavior Surveillance System (YRBSS) to monitor health risk behaviors linked to major causes of morbidity and mortality among US adolescents. The YRBS is an anonymous, school-based survey of high school students, conducted biennially in collaboration with the CDC and state health and education departments. The primary goal of the YRBS is to describe the prevalence of risk behaviors to enhance adolescent health and well-being^20,21^.

Participation in the survey was voluntary, and students completed the self-administered questionnaire during a single class period. The YRBS employs a two-stage cluster sample design to generate a representative sample for each district^20,21^.

### Demographic variables

Students were categorized by sexual orientation based on their responses to the question: ‘Which of the following best describes you’, with those answering ‘Not sure’ being coded as sexual minority students (Supplementary file Table 1). Gender was coded as either male or female based on the students’ responses. In terms of race and ethnicity, students were categorized as White, non-Hispanic, Hispanic, Black, non-Hispanic, or Other race, non-Hispanic. Grade level was coded as either 9th, 10th, 11th, or 12th grade.

### Outcome variables

Cigarette use was measured by asking students their age when they first smoked a whole cigarette. School-based violence victimization was assessed using five measures: absence due to safety concerns, threats or injuries with a weapon on school property, physical fighting on school property, bullying at school, and electronic bullying. Absence due to safety concerns was determined by how many days in the past 30 days students did not attend school due to feeling unsafe. Threats or injuries with a weapon were recorded by asking how many times in the past 12 months, students were threatened or injured with a weapon on school property. Physical fighting was measured by the number of fights on school property in the past 12 months. Bullying at school was assessed by asking if students were bullied at school in the past 12 months. Electronic bullying was measured by asking if students were bullied electronically in the past 12 months (Supplementary file Table 2).

### Inclusion criteria

Students from the 2013, 2015, 2017, 2019, and 2021 Florida YRBS cycles who answered the demographic questions about sexual orientation, race/ethnicity, sex, and grade were included in this analysis. Students who responded to the 2021 question about sexual identity with ‘I do not know what this question is asking’ were excluded from the analysis. Additionally, students who indicated their grade as ‘ungraded/other grade’ were excluded. Students with missing demographic information for any of the variables (sexual orientation, sex, race, ethnicity, or grade) were also excluded.

### Statistical analysis

All statistical analyses were performed using SPSS Version 29.0.0 with the complex sampling module, following CDC guidelines^22,23^. Data were weighted based on values provided by the Florida Department of Health. All outcome measures were recoded and dichotomized as 0 (0 times, 0 days, or no) or 1 (≥1 time, ≥1 day, or yes).

Bivariate analyses (chi-squared tests) were conducted to compare differences in proportions of students who were tobacco users and those who experienced school-based violence. For variables with more than two levels, *post hoc* pairwise comparisons were performed using the least significant difference method to identify specific group differences. Two binary logistic regression models were conducted. The first model evaluated the association between tobacco use (outcome) and school-based violence (predictor), while the second model examined the reverse relationship, with school-based violence as the outcome and tobacco use as the predictor. For both models, covariates included sex, race/ethnicity, sexual orientation, and grade.

The five measures of school-based violence (absence due to safety concerns, threats or injuries with a weapon, physical fighting, bullying at school, and electronic bullying) were collapsed into a single binary variable, where a ‘yes’ to any of the five measures indicated the presence of school-based violence. Adjusted odds ratios (AOR) were calculated with 95% confidence intervals. Reference categories for the logistic regression models were male (for sex), White (for race/ethnicity), heterosexual (for sexual orientation), and 9th grade (for grade).

## RESULTS

### Demographics of study participants

As shown in Table 1, 15.8% of the sample identified as a sexual minority. The racial composition of the same was mixed; 40.6% identified as White, non-Hispanic, 29.6% identified as Hispanic, 23.6% identified as Black, non-Hispanic, and 6.2% identified as another racial minority group. Similarly, about a quarter of the sample represented each grade from 9th grade (26.4%) to 12th grade (23.4%), and about half of the participants were male (50.1%).

**Table 1:** Demographic characteristics of participants, Youth Risk Behavior Survey, Florida, 2013–2021 (N=26510)

*Characteristics*	*Unweighted frequency (n)*	*Weighted percentage (%)*	*95% CI*
**Sex**			
Female	13609	49.9	49.0-50.8
Male	12901	50.1	49.2-51.0
**Grade**			
9th	7769	26.4	24.9-27.9
10th	7188	25.7	24.6-26.8
11th	6431	24.6	23.5-25.6
12th	5122	23.4	22.2-24.6
**Race/Ethnicity**			
**Black**	4970	23.6	21.3-26.1
White	9749	40.6	38.5-42.7
Hispanic	9494	29.6	27.7-31.5
Other	2297	6.2	5.8-6.7
**Sexual Orientation**			
Sexual Minority	4080	15.8	15.3-16.3
Heterosexual	22430	84.2	83.7-84.7

All estimates account for the complex survey design, including strata and cluster variables.

### Prevalence estimates of cigarette smoking

As shown in Table 2, 18.0% of the sample reported cigarette smoking. Of all demographics, sexual minority students (27.3%) had the highest prevalence of cigarette smoking followed by 12th grade students (22.5%) and White students (22.5%).

**Table 2:** Prevalence tobacco use among participants, Youth Risk Behavior Survey, Florida, 2013–2021 (N=26510)

*Characteristics*	*Cigarette use*
**Total**	18.0 (17.3–18.7)
**Sex**	
Female	17.0 (16.1–17.8)
Male	19.0 (18.1–20.0)
**Grade**	
9th	14.3 (13.2–15.4)
10th	16.6 (15.5–17.9)
11th	19.0 (17.9–20.1)
12th	22.5 (21.1–24.1)
**Race/ethnicity**	
Black	10.0 (9.1–11.1)
White	22.5 (21.3–23.7)
Hispanic	18.0 (16.9–19.2)
Other	18.2 (16.3–20.2)
**Sexual orientation**	
Sexual minority	27.3 (25.8–28.8)
Heterosexual	16.3 (15.5–17.1)

Prevalence estimates [% (95% CI)] are based on weighted data. All estimates account for the complex survey design, including strata and cluster variables.

### Prevalence estimates of each measure of school-based violence

The prevalence of school-based violence is shown in Table 3. Among the five types of violence, being bullied at school showed the highest prevalence (14.2%), followed by being electronically bullied (11.8%), and being absent due to safety concerns (10.5%). Of any other demographic, sexual minority students reported the highest prevalence of four measures of school-based victimization, including being bullied at school (24.4%), electronically bullied (22.6%), being absent due to safety concerns (17.3%), and being threatened or injured with a weapon at school (11.2%). Additionally, nearly one-third (29.3%) of students reported experiencing school-based violence. Sex, grade, race/ethnicity and sexual orientation were all significantly associated with experiencing school violence in this sample.

**Table 3:** Prevalence of school-based victimization among participants, Youth Risk Behavior Survey, Florida, 2013–2021 (N=26510)

*Characteristics*	*Absent due to safety concerns*	*Threatened or injured with a weapon at school*	*Physical fighting at school*	*Bullied at school*	*Electronically bullied at school*	*Any school-based violence*
**Total**	10.5 (9.7–11.3)	6.9 (6.5–7.3)	7.0 (6.5–7.5)	14.2 (13.6–14.8)	11.8 (11.3–12.2)	29.3 (28.4–30.2)
**Sex**						
Female	11.6 (10.7–12.5)	5.4 (5.0–5.9)	4.7 (4.3–5.3)	16.9 (16.1–17.9)	15.7 (14.9–16.5)	32.3 (31.2–33.4)
Male	9.4 (8.6–10.3)	8.3 (7.8–8.9)	9.3 (8.6–10.0)	11.5 (10.9–12.1)	7.8 (7.4–8.3)	26.3 (25.3–27.4)
**Grade**						
9th	10.2 (9.2–11.2)	7.5 (6.8–8.3)	9.6 (8.7–10.6)	17.7 (16.7–18.8)	13.5 (12.5–14.6)	33.6 (32.1–35.1)
10th	10.6 (9.5–11.7)	7.1 (6.5–7.8)	7.3 (6.6–8.0)	15.3 (14.6–16.2)	12.2 (11.3–13.1)	30.6 (29.6–31.6)
11th	10.7 (9.5–11.9)	6.5 (5.9–7.3)	6.0 (5.3–6.8)	12.3 (11.4–13.2)	11.0 (10.3–11.7)	27.9 (26.6–29.3)
12th	10.6 (9.6–11.6)	6.3 (5.6–7.0)	4.8 (4.2–5.6)	11.0 (10.1–11.9)	10.1 (9.4–10.9)	24.6 (23.3–26.0)
**Race/ethnicity**						
Black	12.7 (11.3–14.2)	8.1 (7.2–9.1)	10.7 (9.5–12.0)	10.9 (10.0–11.9)	8.1 (7.3–8.9)	29.2 (27.3–31.2)
White	8.3 (7.3–9.3)	6.0 (5.4–6.7)	5.3 (4.7–5.9)	17.0 (16.1–17.9)	14.7 (14.0–15.5)	30.3 (29.0–31.7)
Hispanic	11.7 (10.7–12.7)	6.6 (5.9–7.4)	6.6 (6.0–7.3)	12.6 (11.8–13.5)	10.4 (9.8–11.1)	27.8 (26.6–28.9)
Other	11.0 (9.6–12.7)	9.3 (7.9–10.8)	6.5 (5.4–7.7)	16.0 (14.3–17.9)	12.5 (10.9–14.4)	30.4 (28.0–32.9)
**Sexual orientation**						
Sexual minority	17.3 (15.8–18.9)	11.2 (10.2–12.4)	9.9 (8.8–11.3)	24.4 (22.6–26.2)	22.6 (20.8–24.5)	42.8 (40.8–44.8)
Heterosexual	9.2 (8.5–10.0)	6.1 (5.6–6.6)	6.5 (6.0–7.0)	12.3 (11.8–12.9)	9.7 (9.3–10.1)	26.8 (25.9–27.7)

Prevalence estimates [% (95% CI)] are based on weighted data. All estimates account for the complex survey design, including strata and cluster variables. The variable ‘any school-based violence’ represents a binary outcome where a ‘yes’ response to any of the five measures of school-based violence (absence due to safety concerns, threats or injury with a weapon, physical fighting, bullying at school, and electronic bullying) was coded as a positive response.

### Prevalence of school-based violence types according to tobacco use status

As shown in Table 4, tobacco users were more likely to experience school violence than non-tobacco users. Tobacco users reported higher rates of the following experiences: absent due to safety concerns (χ^2^=206.64; p<0.001), threatened or injured with a weapon at school (χ^2^=334.28; p<0.001), involved in a physical fight at school (χ^2^=370.81; p<0.001), bullied at school (χ^2^=219.07; p<0.001), and electronic bullying (χ^2^=394.04; p <0.001).

**Table 4:** Prevalence of school-based violence types according to tobacco use status, Youth Risk Behavior Survey, Florida, 2013–2021 (N=26510)

*School-based violence types*	*Tobacco use*	*No tobacco use*	*χ^2^*	*p*
Did not go to school because they felt unsafe at school or on their way to or from school	16.1 (14.9–17.4)	8.7 (8.0–9.5)	206.64	<0.001
Were threatened or injured with a weapon on school property	13.1 (12.1–14.2)	5.1 (4.7–5.6)	334.28	<0.001
Were in a physical fight on school property	13.8 (12.6–15.0)	5.2 (4.8–5.6)	370.81	<0.001
Were bullied at school	21.2 (20.0–22.4)	12.5 (11.9–13.1)	219.07	<0.001
Were electronically bullied	20.6 (19.2–21.9)	9.6 (9.2–10.0)	394.04	<0.001

Prevalence estimates [% (95% CI)] are based on weighted data. All estimates account for the complex survey design, including strata and cluster variables. Chi-squared tests were used to assess differences between tobacco users and non-users across various forms of school-based violence. For categorical variables with more than two levels, post hoc pairwise comparisons were conducted using the least significant difference (LSD) method.

### Logistic regression model results

Logistic regression models were used to investigate whether school-based violence was associated with tobacco use (Table 5). The direct effects observed indicated tobacco use was associated with school violence (AOR=2.04; 95% CI: 1.91–2.19) while controlling for sex, grade and race/ethnicity. Compared to the reference group of 9th grade students, 12th grade students were the most likely subgroup to report tobacco use (AOR=1.99; 95% CI: 1.78–2.23). Similarly, compared to heterosexual students, sexual minority students (AOR=1.91; 95% CI: 1.75–2.09) were more likely to report tobacco use.

**Table 5:** Logistic regression models, Youth Risk Behavior Survey, Florida, 2013–2021 (N=26510)

*Direct effect*	*AOR (95% CI)*	*p*
**Tobacco use**		
School-based violence	2.04 (1.91–2.19)	<0.001
No experience of school violence	®	
Sexual minority status	1.91 (1.75–2.09)	<0.001
Heterosexual	®	
Female	0.75 (0.69–0.81)	<0.001
Male	®	
9th grade	®	
10th grade	1.26 (1.13–1.41)	<0.001
11th grade	1.53 (1.37–1.72)	<0.001
12th grade	1.99 (1.78–2.23)	<0.001
White	®	
Black	0.38 (0.33–0.43)	<0.001
Hispanic	0.76 (0.69–0.83)	<0.001
Other race	0.74 (0.65–0.84)	<0.001
**School-based violence**		
Tobacco use	2.13 (1.99–2.29)	<0.001
No tobacco use	®	
Sexual minority status	1.84 (1.68–2.02)	<0.001
Heterosexual	®	
Female	1.24 (1.17–1.32)	<0.001
Male	®	
9th grade	®	
10th grade	0.80 (0.74–0.87)	<0.001
11th grade	0.68 (0.62–0.74)	<0.001
12th grade	0.53 (0.49–0.58)	<0.001
White	®	
Black	0.98 (0.87–1.11)	0.754
Hispanic	0.89 (0.81–0.96)	0.006
Other race	1.003 (0.87–1.16)	0.96

The analysis was conducted using two binary logistic regression models. In the first model, school-based violence served as the outcome variable, with tobacco use as the predictor, while controlling for sex, race/ethnicity, sexual orientation, and grade. In the second model, tobacco use was the outcome, with school-based violence as the predictor, controlling for the same covariates. Adjusted odds ratios (AOR) are presented with 95% confidence intervals. Adjustments for multiple comparisons were made using the least significant difference (LSD) method. ® Reference categories.

Tobacco use was also a significant predictor of school-based violence (AOR=2.13; 95% CI: 1.99–2.29). School-based violence was more likely to be experienced by sexual minority students compared to their heterosexual peers (AOR=1.84; 95% CI: 1.68–2.02) and female students (AOR=1.24; 95% CI: 1.17–1.32). Compared to the reference group of 9th grade students, 12th grade students were the least likely to experience school violence (AOR=0.68; 95% CI: 0.62–0.74), followed by 11th grade students (AOR=0.68; 95% CI: 0.62–0.74) and finally 10th grade students (AOR=0.80; 95% CI: 0.74–0.87). When compared to the reference group of White students, Hispanic students were also the least likely to experience any school-based violence (AOR=0.89; 95% CI: 0.81–0.96).

Additionally, an interaction between school-based violence and sexual minority status was tested to explore whether the compounded effect of these factors predicted tobacco use. This model controlled for sex, race/ethnicity, sexual orientation, and grade, consistent with the other models. The interaction term was not statistically significant (p=0.499), suggesting that the relationship between school-based violence and tobacco use remained consistent across sexual minority status.

## DISCUSSION

This study found significant levels of school-based violence victimization among adolescents, with 29.3% of students reporting at least one form of victimization. The most common forms were bullying at school (14.2%) and electronic bullying (11.8%). Sexual minority students experienced higher rates of violence compared to their heterosexual peers. This increased likelihood of experiencing victimization among sexual minority students is consistent with minority stress theory, which posits that individuals with marginalized identities are more likely to encounter stressors, including discrimination and victimization, as a direct result of their minority status. Sexual minority students are more vulnerable to school-based violence due to societal stigma and discriminatory behaviors that persist in school environments, making them a particularly high-risk group for both victimization and subsequent health-related behaviors like tobacco use.

The relationship between school-based violence and tobacco use was notable, with students who reported any form of victimization having higher odds of using tobacco. Sexual minority students showed a higher prevalence of both victimization and tobacco use, underscoring the compounded risks faced by this group. While the study tested for an interaction between sexual minority status and school-based violence to determine whether the compounded effect of these factors predicted tobacco use, the interaction was not statistically significant (p=0.499). This suggests that, although sexual minority students experience higher levels of both violence and tobacco use independently, the combined effect of these two factors did not significantly amplify the likelihood of tobacco use in this sample. Nonetheless, the findings emphasize that by the very experience of being a sexual minority, one is more likely to experience victimization, and thus indirectly more vulnerable to engaging in health-risk behaviors like tobacco use.

These results align with previous research indicating that violence exposure, such as bullying, significantly increases the risk of substance use among adolescents^8,12-14^. Studies consistently show that adolescents who experience bullying or other forms of school-based violence are more likely to engage in tobacco use as a coping mechanism. For example, Duangchan et al.^13^ found a similar association between school-based victimization and tobacco use, particularly among sexual minority students, who faced elevated risks of both victimization and substance use. This pattern holds true across various contexts, including international studies that have examined the link between bullying and tobacco use. A study conducted by Pichel et al.^14^ in Spain found that bullying and cyberbullying were strong predictors of substance use among youth, suggesting that the mechanisms driving this relationship are not limited to the US context. However, while international studies provide valuable insights, cultural and policy differences may influence the magnitude of these associations. For instance, countries with stronger anti-bullying policies or more inclusive environments for sexual minorities may observe lower rates of both victimization and tobacco use.

National survey data in the US, including findings from the Monitoring the Future (MTF) survey and the Youth Risk Behavior Survey (YRBS), have consistently demonstrated the role of school-based violence and victimization in shaping adolescent substance use^13,19,24-26^. Wu et al.^25^ found that students who experienced victimization reported significantly higher rates of vaping and tobacco use compared to their non-victimized peers, highlighting school-based violence as a significant factor in adolescent substance use behaviors. Barbero et al.^26^ similarly observed that various forms of bullying, including at-school and electronic victimization, were associated with increased likelihoods of substance use among middle school students, with adjusted prevalence ratios showing strong associations across behaviors like alcohol, marijuana, and electronic vapor product use^26^. Such patterns suggest that exposure to bullying from an early age is linked to an increased risk of substance use, with effects that persist into high school and influence tobacco use patterns. Consistent with these findings, the current study observed higher odds of tobacco use among students who experienced school-based violence, with sexual minority students showing particularly high rates of both victimization and tobacco use. Although the interaction between sexual minority status and school-based violence was not statistically significant, the elevated rates of tobacco use among both victimized and sexual minority students reflect similar patterns in previous research, reinforcing the compounded vulnerability of this population.

### Implications

This study emphasized the significant link between school-based violence and tobacco use among adolescents, particularly for those who faced discrimination and harassment based on their sexual orientation. The findings had critical implications in the context of Florida’s ‘Don’t Say Gay’ bills and other anti-LGBTQ policies in schools. These policies, which restricted discussions of sexual orientation and gender identity in educational settings, may have intensified the negative experiences of LGBTQ students, making them more susceptible to bullying and other forms of school-based violence^17,27-29^. As a result, these policies could inadvertently lead to increased tobacco use as students use smoking as a coping mechanism in hostile environments^24^. The findings underscore the necessity for schools to create inclusive and supportive environments for all students, regardless of their sexual orientation or gender identity. Additionally, policymakers and educators should consider the broader impacts of such legislation on student well-being and substance use patterns, as fostering inclusive and affirming school environments is crucial for the health and safety of all students.

### Strengths and limitations

This study has several notable strengths, including a large and diverse sample size and a comprehensive dataset spanning from 2013 to 2021, which enhances the reliability and generalizability of the findings. With 26510 participants, the analysis was able to detect statistically significant results and draw meaningful conclusions across various demographic subgroups. The use of self-reported data for demographic variables, particularly sexual orientation, added depth and accuracy to the analysis, as self-identification often provided more reliable and nuanced information compared to school-provided records.

However, several limitations should be acknowledged. The cross-sectional nature of the data limited the ability to establish causal relationships between variables. Longitudinal research is needed to determine the direction of causality and the long-term effects of school-based violence on tobacco use patterns. Additionally, the reliance on self-reported data may have introduced social desirability bias and inaccuracies in participants’ recall. Another limitation was the study’s focus on a specific geographical area, such as Florida, which may not be generalizable to adolescents in other regions with different cultural, socioeconomic, and policy contexts. Furthermore, the study may not account for all potential confounding variables, such as other forms of trauma or environmental factors that could influence the observed relationships. Future research should address these limitations by incorporating longitudinal designs, using more comprehensive data sources, and examining additional factors that may impact the associations studied. Despite these limitations, the study contributes to the understanding of the complex relationship between school-based violence, demographic characteristics, and tobacco use in adolescents.

## CONCLUSIONS

This study explored the factors influencing tobacco use patterns among a racially diverse group of adolescents in Florida. The findings reveal that tobacco use is particularly prevalent among adolescents, posing significant public health challenges. School-based violence emerged as a notable contributor, shaping adolescents’ behaviors and trajectories. The high prevalence rates underscore the need for targeted intervention strategies, especially for vulnerable student populations. Tobacco use often served as a coping mechanism for adolescents facing adversity, intertwining with experiences of violence and perpetuating adverse outcomes. Therefore, interventions should address both tobacco use and the underlying causes of violence and discrimination within educational settings. Recognizing the heightened risks based on sexual orientation and exposure to school-based violence, interventions must foster safer environments for all students, promoting positive youth development and societal well-being. The findings emphasize creating inclusive and supportive school environments to mitigate these risks. By addressing and reducing school-based violence, schools can play a crucial role in reducing tobacco use among adolescents and enhancing their overall health and well-being.

## Supplementary Material



## Data Availability

The data supporting this research are available from the following link: https://www.floridahealth.gov/statistics-and-data/survey-data/index.html
