# Supplementary material for: Understanding tobacco use disparities among Florida adolescents: The impact of sexual minority status and school-based violence victimization
Source: Tob Prev Cessat. 2024 Nov 20;10:10.18332/tpc/195288. doi: 10.18332/tpc/195288 (PMC11577446; doi:10.18332/tpc/195288)
Supplement: Supplementary file 1 [file TPC-10-55-s1.pdf]

**Supplementary Table 1**. Demographic characteristics of study participants, Youth Risk Behavior Survey, Florida, 2013–2021

| Demographic Variable | Question |
|---|---|
| Sexual Orientation | Which of the following best describes you?<br>A. Heterosexual<br>B. Gay or lesbian<br>C. Bisexual<br>D. Not sure |
| Sex | What is your sex? |
| Race | What is your race? |
| Ethnicity | Are you Hispanic or Latino? |
| Grade | In what grade are you? |

**Supplementary Table 2.** Youth Risk Behavior Survey Questions, Youth Risk Behavior Survey, Florida, 2013–2021

| Measure | Question |
|---|---|
| **School-related violence victimization** | |
| Absent due to safety concerns | During the past 30 days, how many days did you not go to school because you felt you would be unsafe at school or on your way to school? |
| Threatened or injured with a weapon at school | During the past 12 months, how many times has someone threatened or injured you with a weapon such as a gun, knife or club on school property? |
| Physical fighting at school | During the past 12 months, how many times were you in a physical fight on school property? |
| Bullied at school | During the past 12 months, have you ever been bullied at school? |
| Electronically bullied | During the past 12 months, have you ever been electronically bullied at school? (Count being bullied through email, chat rooms, instant messaging, websites, or texting.) |
| **Substance use** | |
| Lifetime cigarette use | How old were you when you smoked a whole cigarette for the first time? |

©2024 Gumbs C.M. et al.
